# Rapid identification of acrolein scavengers from *Anoectochilus roxburghii* and evaluation of their representative antiglycation and antioxidant activities

**DOI:** 10.1016/j.fochx.2026.104585

**Published:** 2026-10-07

**Authors:** Guo Dong Zhuang, Yong Pei Tang, Rui Long Zhang, Hang Hong Lo, Lin Lin Song, Xiao Yun Yun, Jian Hui Wu, Cai Ren Wang, Hui Miao Wang, Meng Han Liu, Zhi Kang Wang, Vincent Kam Wai Wong, Betty Yuen Kwan Law

**Affiliations:** a Dr. Neher's Biophysics Laboratory for Innovative Drug Discovery, The State Key Laboratory of Mechanism and Quality of Chinese Medicine, Faculty of Chinese Medicine, Macau University of Science and Technology, Macau, China; b Macau University of Science and Technology Zhuhai MUST Science and Technology Research Institute, China

**Keywords:** Acrolein, ACR-incubating UHPLC-DAD-HRMS/MS, Food safety, Natural ACR scavengers, Anti-glycation

## Abstract

Abstract

Acrolein (ACR), a highly reactive α,β-unsaturated carbonyl compound generated during thermal food processing and lipid peroxidation, contributes to carbonyl stress and oxidative protein modification. In this study, a reaction-guided UHPLC-DAD-HRMS/MS strategy was developed to screen natural ACR scavengers from *Anoectochilus roxburghii* (*A. roxburghii*). Eight major ACR-reactive compounds were identified, and narcissoside (NAR) was selected for further validation due to its relatively high abundance and confirmed ACR reactivity. NAR directly trapped ACR *in vitro* through mono-, di-, and tri-adduct formation, while *in vivo* fecal analysis suggested that its metabolite isorhamnetin (ISO) contributed to ACR trapping. NAR also attenuated protein modification in ACR–BSA, MGO–BSA, Glu–BSA, and Fru–BSA models. Furthermore, low-dose subacute ACR exposure showed that NAR and ISO improved serum carbonyl stress, oxidative stress, and inflammatory markers. These findings identify NAR as a representative ACR-trapping constituent of *A. roxburghii*.

## Highlights

• A reaction-guided strategy enabled rapid screening of acrolein scavengers.
• Eight acrolein-reactive compounds were identified from *Anoectochilus roxburghii*.
• Narcissoside inhibited AGEs formation and protein glycoxidation.
• NAR and ISO alleviated ACR-induced carbonyl stress and inflammation.
• Potential application in mitigating carbonyl stress in food systems.

## Graphical abstract

**Figure:**
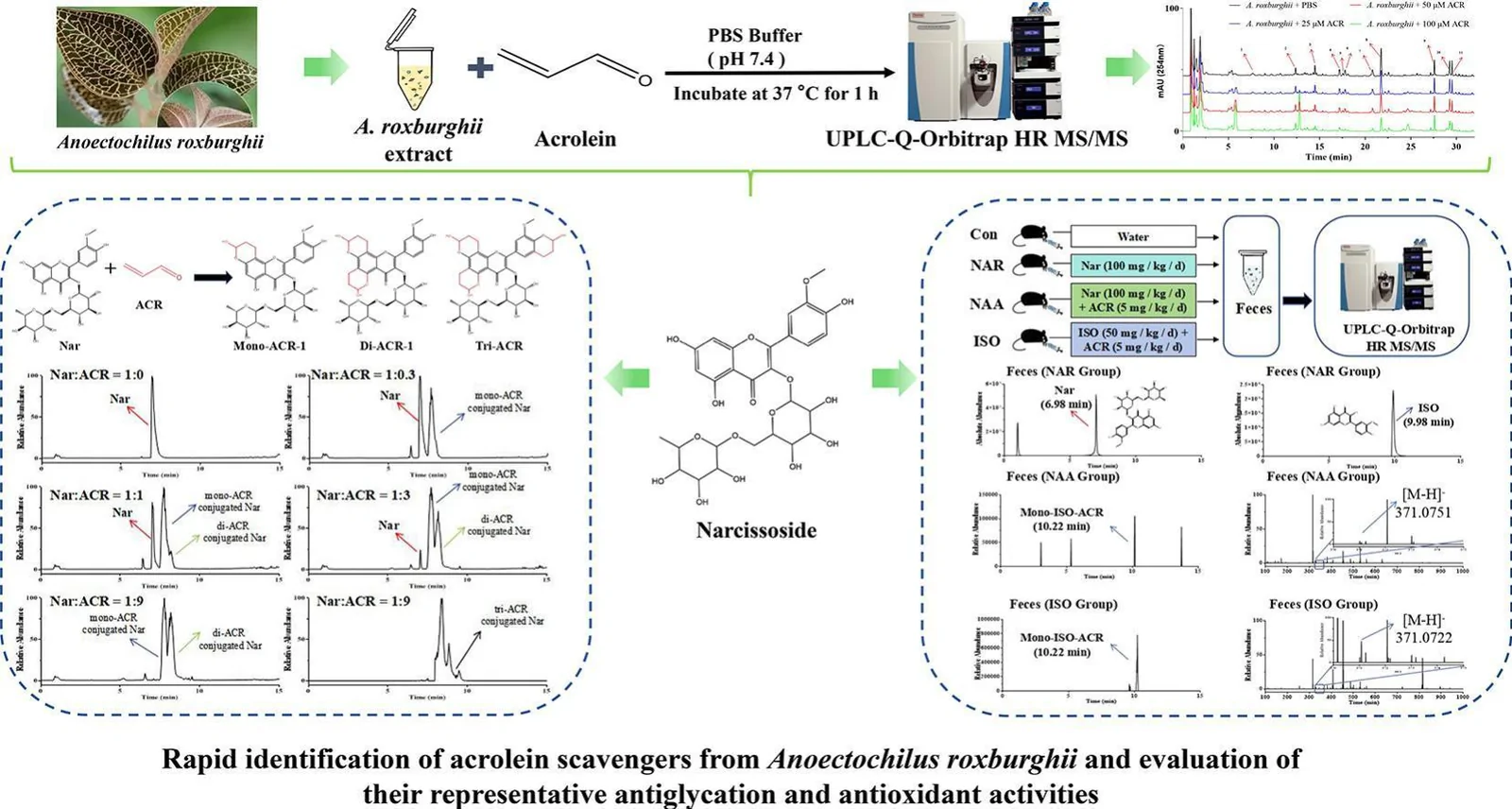

## 1 Introduction

ACR is a highly reactive α,β-unsaturated carbonyl compound that poses significant toxicological concerns in food and biological systems (Du et al., 2024). The International Agency for Research on Cancer has listed this compound as a Group 3 carcinogen and it is considered as an independent reactive carbonyl species that contributes to carbonyl stress, protein carbonylation, and food quality deterioration (Zhang et al., 2024). Owing to its electrophilic carbonyl and vinyl functional groups, ACR readily reacts with nucleophilic sites in biological macromolecules, including proteins, nucleic acids, and phospholipids, leading to the formation of stable adducts and cross-linked product (Chuang et al., 2025; Wang, Tong, et al., 2025). Such interactions elevate the risks of carcinogenic and teratogenic effects (Reiter et al., 2025). These reactions contribute to cytotoxicity, mutagenicity, and tissue dysfunction, and have been implicated in the progression of chronic diseases such as Alzheimer's disease (AD), Parkinson's disease (PD), diabetes, and cardiovascular disorders (Augustine et al., 2025; Wang, Ou, et al., 2025). Studies have revealed that ACR levels in brain tissue, cerebrospinal fluid, and plasma are markedly elevated in individuals with mild cognitive impairment (MCI) and AD compared to healthy controls (Sanotra et al., 2023; Xie et al., 2025). Increasing evidence further indicates that ACR can promote the formation of AGEs, Nε-carboxymethyllysine (CML) and advanced lipid oxidation end products (ALEs), which are closely associated with oxidative stress, inflammation, and neurodegeneration (Wang et al., 2019; Yang, Li, et al., 2025).

ACR is widely present in thermally processed foods, including fried, baked, and roasted products, and can also be generated endogenously during lipid peroxidation and amino acid metabolism (Gu et al., 2025). Due to its high reactivity and toxicity, chronic exposure poses significant health risks. Therefore, developing safe and effective ACR scavengers to reduce its accumulation in the body is considered a promising strategy for preventing and alleviating ACR-related chronic diseases. To date, various approaches have been employed to mitigate the safety risks posed by ACR in processed foods (Gu, Wang, Si, et al., 2025; Sugimoto et al., 2021). Among these, the use of natural ACR scavengers is regarded as one of the most favorable strategies. Examples include certain flavonoids (*e.g.*, Hesperidin), histidine (*e.g.*, Ergothioneine), Alkaloidand (*e.g.*, Synephrine) and Phenolic acids (*e.g.*, Octyl gallate), most of which have demonstrated effective ACR scavenging capacity both *in vitro* and *in vivo* (Gu, Wang, Wen, et al., 2025; Jia, Gu, et al., 2023; Qiu et al., 2022; Zhong et al., 2024). However, many currently available scavengers exhibit limitations, including poor stability, low bioavailability, and undesirable effects on food quality, which restrict their practical application. Consequently, the identification of novel and effective natural ACR scavengers remains highly desirable.

A key challenge in this field is the lack of rapid and efficient screening techniques for identifying ACR scavengers directly from complex natural matrices. Conventional bioactivity-guided isolation approaches are often time-consuming and labor-intensive. In previous research, a novel analytical method based on 2,2′-azobis(2-methylpropanimidamide) dihydrochloride (AAPH) incubation combined with HPLC-DAD-HRMS/MS was developed, allowing efficient and high-throughput screening of antioxidant compounds directly within natural product extracts (Zhuang et al., 2023). Furthermore, to our knowledge, there have been no reports describing a screening strategy that integrates pre-column reaction with ACR and UHPLC-DAD-MS^2^ for the direct detection and characterization of naturally occurring ACR scavengers.

*Anoectochilus roxburghii* (*A. roxburghii*) is a prominent medicinal and edible plant species widely distributed in southern China, whose utilization has gained increasing popularity in recent years (Li, Ran, et al., 2026; Yang, Sun, et al., 2025). Highly regarded in various Asian traditional medicine systems, particularly in China and Japan, this herb is recognized for its diverse therapeutic benefits. It has traditionally been used for its anti-inflammatory, hepatoprotective, and antioxidant activities and is considered a valuable source of bioactive phytochemicals, particularly flavonoids and phenolic compounds (Li, Cheng, et al., 2026; Wang et al., 2020; Wu et al., 2024). These characteristics suggest that AR may represent a promising source of natural ACR scavengers.

Therefore, the objective of this study was to develop a rapid and reaction-guided UHPLC-DAD-HRMS/MS screening strategy for the direct identification of ACR scavengers from natural product extracts. This method is based on monitoring chromatographic peak area changes following incubation with ACR, allowing rapid localization and structural characterization of reactive compounds. Using AR as a model system, natural ACR scavengers were successfully identified. Furthermore, narcissoside was selected as a representative compound, and its ACR-trapping capacity and anti-glycation effects were further evaluated through *in vitro* and *in vivo* experiments. This study provides a practical and efficient approach for the rapid discovery of natural ACR scavengers and highlights the potential of plant-derived compounds for reducing ACR-related food safety risks.

## 2 Material and methods

### 2.1 Reagents

NAR and Isorhamnetin (ISO) was provided by Chengdu Pryfa Technology Development Co., Ltd. (Chengdu, China). Glucose was supplied by Shanghai Yuanye Biotechnology Co., Ltd. (Shanghai, China). Fructose obtained from Beijing Solebo Company Co., Ltd. (Beijing, China). Bovine serum albumin was purchased from Guangzhou Ruishu Biotechnology Co., Ltd. (Guangzhou, China). Methylglyoxal (MGO) was obtained from Aladdin Industrial Corporation Co., Ltd. (Shanghai, China). ACR was obtained from Sigma Chem Co. (St. Louis).

### 2.2 Preparation of *A. roxburghii*

*A. roxburghii*. was supplied by Kangmei Pharmaceutical Co., Ltd. (Guangzhou, China). An accurately weighed sample (1 g) was extracted with 30 mL distilled water using ultrasonic-assisted extraction for 30 min. Following extraction, centrifugation was conducted at 10,000 rpm for 10 min, and the supernatant was collected and filtered through a 0.22 μm membrane before instrumental analysis.

### 2.3 Screening of ACR scavengers by ACR-incubating UHPLC-DAD-HR MS/MS

The 1 mL *A. roxburghii*. crude extract and an equal volume of ACR solutions in PBS (100 mM, pH 7.4) of different concentrations (1 mL, 0, 25, 50 and 100 μM, final concentration, similarly hereinafter) were added to a 5 mL screw-cap glass reaction vial and reacted in a shaking at 70 rpm for 37 °C respectively. The crude extract of *A. roxburghii*. was incubated with ACR solutions at different concentrations for 1 h in order to optimize the appropriate ACR concentration for subsequent experiments. This procedure aimed to investigate the effect of ACR levels on the decrease in chromatographic peak areas and to evaluate the ACR scavenging potential of individual components based on their peak reduction percentages. After incubation, the reaction mixtures were filtered through a 0.22 μm membrane, and the resulting solutions were immediately analyzed using UHPLC (Fig. 1A). PBS-treated samples were simultaneously prepared as blank controls for comparison. Chromatographic peak areas were calculated from UHPLC-DAD chromatograms recorded at 254 nm. ACR-reactive compounds were screened by comparing the peak areas of major constituents before and after ACR incubation. The peak reduction percentage was calculated according to Formula (1), and this parameter was used to estimate the ACR scavenging capacity of the screened compounds.

(1) $$\text{Peak reduction rate}\;\left(\%\right)=\left(1-U_{\text{after}}/U_{\text{before}}\right)\times 100$$

Where U_after_ = the peak area value of extracts after ACR-Incubating, U_before_ = the peak area value of extracts before ACR-Incubating.

**Fig. 1:**
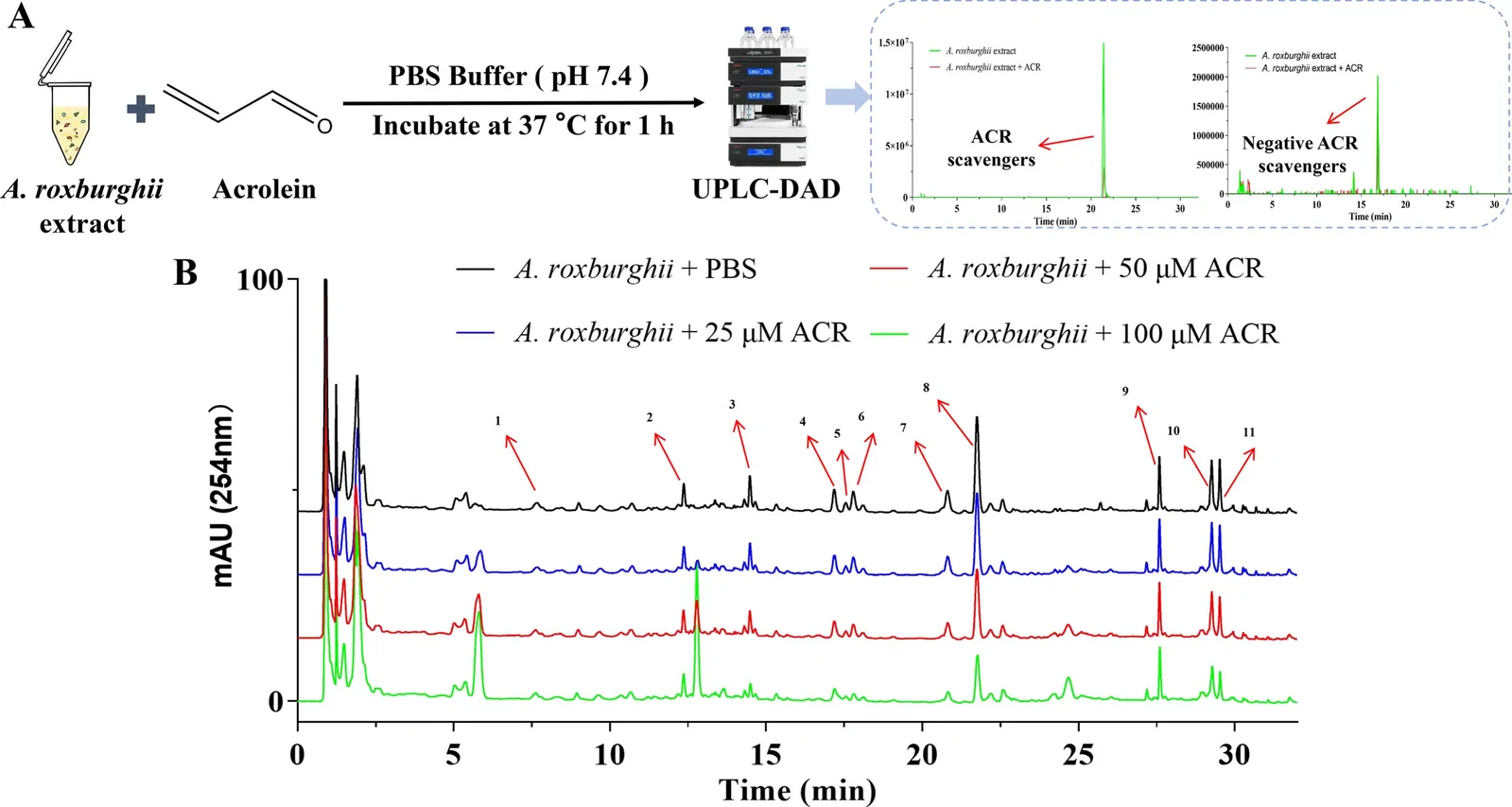
The ACR-incubation HPLC-DAD-HR MS/MS screening of natural ACR scavengers directly from *A. roxburghii*. (A) The experimental design of ACR-incubation HPLC-DAD-HR MS/MS. (B) The effect of the ACR solution at different concentrations for the screening of ACR scavengers in the chromatogram. (1. Glucosyringic acid, 2. Glucosylrutin, 3. Rutin, 4. Kaempferol 3,7-diglucoside, 5. Isoquercitrin, 6. Quercimeritrin, 7. Kaempferol-rutoside, 8. NAR, 9. Feruloylquinic acid methyl ester, 10. ISO, 11. Emodin).

### 2.4 UHPLC-DAD-HR MS/MS analysis for *A. roxburghii*

A Dionex 3000 Ultimate HPLC interfaced with an auto-sampler and a Q-Exactive Orbitrap HRMS/MS. HPLC separations were performed on an Acquity UPLC BEH C18 (2.1 × 100 mm, 1.7 μm) that was run at 35 °C. The mobile phase was composed of: acidified water (0.1% formic acid) (eluent A) in water, and Acetonitrile (eluent B). The injection volume was 3 μL and the elution flow rate was 0.3 mL/min. The elution parameters were: 0–2 min, 2%–2% B; 2–8 min, 2%–6% B; 8–11 min, 6%–13% B; 11–19 min, 13%–15% B; 19–22 min, 15%–20% B; 22–25 min, 20%–30% B; 25–29 min, 30%–50% B; 29–31 min, 50%–60% B; 31–34 min, 60%–80% B.

HRMS/MS analysis was performed with a heated electrospray ionization source in negative ionization mode. The mass parameters were set as follows: auxiliary gas flow, 15 arb; sheath gas flow, 45 arb; spray voltage, 3500 V; mass scan range, *m*/*z* 100–1000; auxiliary gas heater temperature, 100 °C; capillary temperature, 350 °C.

### 2.5 Quantification of representative ACR-reactive compounds by LC-QQQ-MS/MS

The contents of representative ACR-reactive compounds in *A. roxburghii* extract were determined using an LC-QQQ-MS/MS system equipped with an Agilent 6460 triple quadrupole mass spectrometer. Standard solutions of rutin, NAR and ISO were prepared at appropriate concentrations to construct external calibration curves. The *A. roxburghii* extract was dissolved in methanol/water, ultrasonicated, centrifuged, and filtered through a 0.22 μm membrane prior to analysis. Quantification was performed using the external standard method, and the contents of the three compounds were expressed as μg/g of *A. roxburghii* extract. All samples were analyzed in triplicate. The optimized MRM transitions, retention times and collision energies for rutin, NAR, and isorhamnetin are summarized in Table S1.

### 2.6 Identification of the NAR-ACR adducts using UHPLC−orbitrap MS/MS analysis

NAR (1 mM) was incubated with ACR (0.3 mM, 1 mM, 3 mM and 9 mM) in 37 °C for 1 h. Subsequently, the resulting adducts were analyzed *via* a UHPLC-Q-Orbitrap HRMS/MS System (Thermo, USA) equipped with an electrospray ionization (ESI). The above samples underwent filtration through a 0.22 μm syringe nylon membrane. Afterward, 2 μL of sample was separated in Acquity UPLC BEH C18 (2.1 × 100 mm, 1.7 μm). The mobile phases were 0.1% formic acid (solvent A) and acetonitrile (solvent B). The elution gradient program was as follows: 5–5% B from 0 to 3 min, 10–90% B from 3 to 9 min, 90–90% B from 9 to 11 min, with 2 min of equilibration. The flow rate and column temperature were 0.3 mL/min and 30 °C, respectively.

It should be noted that these ACR concentrations (0.3–9 mM) were not intended to simulate physiological exposure levels or actual concentrations generated during food processing. Instead, they were used as analytical reaction conditions to enrich adduct signals and to determine whether NAR could form mono-, di-, and tri-ACR adducts. As this experiment was performed in a cell-free system, cytotoxicity or animal toxicity was not a limiting factor in the selection of ACR concentrations.

### 2.7 Scavenging ACR by NAR in mice

Ethics statement: All animal experiments conducted in this study were performed in strict accordance with relevant ethical guidelines and were formally approved by the Institutional Animal Care and Use Committee of Macau University of Science and Technology (MUST-FDCT-20251021001). Furthermore, this study was conducted in accordance with the National Research Council Guide for the Care and Use of Laboratory Animals. Male C57BL/6 mice (8 weeks old, 20–24 g) were obtained from the Guangdong Medical Laboratory Animal Center. After a 7-day acclimatization period, the mice (*n* = 24) were randomly assigned to four groups (*n* = 6 per group): control group (Con, received 0.1% dimethyl sulfoxide, DMSO), NAR group (NAR, 100 mg/kg), NAR + ACR group (NAA, 100 mg/kg), and ISO + ACR group (ISO, 50 mg/kg). For the short-term *in vivo* ACR-trapping experiment, mice were fasted for 12 h before administration, with free access to water. The fasting procedure was performed to reduce the interference of gastric and intestinal contents with the *in vivo* trapping reaction between NAR/ISO and ACR. NAR or ISO was administered by oral gavage, followed by oral administration of ACR solution 30 min later. The ACR dose used in this experiment was 5 mg/kg body weight. Fecal samples were collected over 24 h and stored at −80 °C. At the end of the experiment, mice were anesthetized with isoflurane and then euthanized by cervical dislocation to minimize suffering. All procedures were performed after completion of the 24 h fecal collection period.

### 2.8 LC-MS/MS analysis for ACR adducts *in vivo*

Two hundred milligrams of dried feces from each group were homogenized in 1 mL of acetonitrile for 60 s, followed by ultrasonic extraction for 60 min. The suspension was centrifuged at 14,000 ×*g* for 10 min at 4 °C. This extraction procedure was repeated twice. The combined supernatants were evaporated under nitrogen, and the residue was reconstituted in 100 μL of acetonitrile, then centrifuged at 11,000 rpm for 15 min at 4 °C. Finally, 10 μL of the resulting supernatant was analyzed by UPLC-Q-Orbitrap HRMS/MS.

### 2.9 Determination of anti-glycation capability by using ACR–BSA, MGO–BSA, Glu–BSA, and Fru–BSA models

The anti-glycation capabilities of NAR were assessed using four different models: BSA-ACR, BSA-MGO, BSA-Glu and BSA-Fru (Błaszczak et al., 2020; Eze & Tola, 2020; Wang et al., 2019). Briefly, BSA was dissolved in PBS (pH 7.4) with a final concentration of BSA at 30 mg/ml and incubated with ACR (1.5 mM) and NAR. For the BSA-Glu model, a control solution was prepared by dissolving 1 g of BSA and 14.4 g of d-glucose in 100 mL of PBS. This resulted in a solution with a concentration of 10 mg/mL BSA and 0.8 M d-glucose. In the BSA-Fru model, a control solution was prepared by dissolving 1 g of BSA and 45.05 g of fructose in 100 mL of PBS. This resulted in a solution with a concentration of 10 mg/mL BSA and 2.5 M fructose. In the BSA-MGO model, a control solution was prepared by dissolving 0.1 g of BSA and 90.9 μL of MGO (40% solution) in 100 mL of PBS. This resulted in a solution with a concentration of 1 mg/mL BSA and 5 mM MGO. To evaluate the anti-glycation capabilities of NAR, test solutions were prepared. Each test solution contained 2.25 mL of control solution and, where indicated, 0.75 mL of NAR (in PBS, pH 7.4). To keep the final reaction volume consistent for all conditions, an additional 0.75 mL of PBS (pH 7.4) was added to the samples without NAR. All solutions were incubated at 37 °C for 7 days, with 0.2 g/L NaN₃ as a preservative. Aminoguanidine (AG, 0.5 and 1.0 mM), a classical inhibitor of AGE formation, was included as a positive control. Following the 7-day incubation period, fluorescence intensity was quantified at an excitation wavelength of 370 nm and an emission wavelength of 440 nm using a Spark™ Multimode Microplate Reader. The inhibition rate of AGEs or protein oxidation products was determined using the following Formula (2):

(2) $$\text{Inhibition}\;\left(\%\right)=\left(1–\text{Fluorescence intensity of test solution with treatment}/\text{Fluorescence intensity of control}\right)\times 100$$

### 2.10 Determination of protein glyoxidation products

Fluorescence intensity of protein glycoxidation products was measured at specific excitation/emission wavelengths: dityrosine (λ_ex_ 330 nm / λ_em_ 415 nm), kynurenine (λ_ex_ 365 nm / λ_em_ 480 nm), and N′-formyl-kynurenine (λ_ex_ 325 nm / λ_em_ 434 nm). The inhibition rate of AGEs or protein oxidation products was determined using the Formula (2):

### 2.11 Analysis of the antioxidant property

The antioxidant capacity of NAR was evaluated from the scavenging DPPH free radical, and ABTS free radical, examined using their respective assay kits, and vitamin C (VC) (1.25 mM) was employed as the positive control. Scavenging activity of the DPPH free radical, and ABTS free radical could be obtained using the following Formula (3) and Formula (4):

(3) $$\text{DPPH scaverging rate}\;\left(\%\right)=\left[1-\left(A_{\text{sample}}-A_{\text{control}}\right)\right]/A_{\text{blank}}\times 100$$

(4) $$\text{ABTS scaverging rate}\;\left(\%\right)=\left(A_{\text{blank}}-A_{\text{control}}\right)/A_{\text{blank}}\times 100$$

### 2.12 Low-dose subacute ACR exposure and serum biomarker analysis

To further evaluate the biological effects of NAR and ISO under repeated low-dose ACR exposure, a low-dose subacute animal experiment was performed. Both the short-term *in vivo* ACR-trapping experiment and the newly added low-dose subacute ACR exposure experiment were covered by the same animal ethics approval and conducted in accordance with the National Research Council Guide for the Care and Use of Laboratory Animals. Male C57BL/6 J mice were acclimatized for 7 days under standard laboratory conditions with free access to food and water. After acclimatization, the mice were randomly divided into six groups, with eight mice in each group: control group, ACR model group, ACR + low-dose NAR group (NAL), ACR + high-dose NAR group (NAH), ACR + low-dose ISO group (ISL), and ACR + high-dose ISO group (ISH).

Mice in the ACR model group were orally administered ACR at a dose of 3.5 mg/kg body weight once daily for 14 consecutive days (Chen et al., 2022). In the NAL and NAH groups, mice were orally administered NAR at doses of 40 and 80 mg/kg body weight, respectively, before ACR exposure. In the ISL and ISH groups, mice were orally administered ISO at doses of 40 and 80 mg/kg body weight, respectively, before ACR exposure. Mice in the control group received the corresponding vehicle. This model was designed as a repeated low-dose subacute ACR exposure model to evaluate serum biochemical responses, rather than as a direct simulation of daily human dietary exposure.

At the end of the 14-day treatment, mice were anesthetized with isoflurane, and blood samples were collected. After blood collection, all mice were euthanized by cervical dislocation under isoflurane anesthesia to minimize suffering. Blood samples were allowed to clot at room temperature and then centrifuged to obtain serum. The serum samples were stored at −80 °C until biochemical analysis. To assess ACR-induced serum glycation/carbonyl stress, oxidative stress, and inflammatory responses, serum levels of AGEs, CML, malondialdehyde (MDA), superoxide dismutase (SOD), IL-1β, TNF-α and IL-6 were determined. AGEs and CML were measured as representative glycation/carbonyl stress-related markers. MDA was used as an indicator of lipid peroxidation, whereas SOD activity was measured to evaluate antioxidant defense capacity. IL-1β, TNF-α, and IL-6 were measured as inflammatory cytokines.

All serum biomarkers were determined using commercial assay kits according to the manufacturers' instructions. The absorbance or fluorescence intensity was measured using a microplate reader at the recommended wavelength for each assay. The results were expressed according to the corresponding kit protocols.

### 2.13 Statistical analysis

The results were presented as means ± standard deviations. IC50 values were calculated by nonlinear regression analysis using a dose-response inhibition model. Data was conducted using ANOVA followed by Duncan's multiple tests with IBM SPSS Statistics 20 (SPSS, Inc., Chicago, IL, USA). Significance was established at *p* < 0.05.

## 3 Results and discussion

### 3.1 Screening of the ACR scavengers by ACR-incubating UPLC-DAD

ACR is widely present in food products and environmental sources encountered in daily life. The World Health Organization has established a tolerable oral intake level of 7.5 mg/kg body weight per day for ACR (Liu et al., 2023). Consequently, minimizing ACR exposure from dietary sources and within the human body is important for protecting public health. Structurally, ACR is classified as an electrophilic α,β-unsaturated aldehyde, and its highly reactive carbonyl group can interact with nucleophilic compounds (such as hesperidin) to generate corresponding adducts. Therefore, these compounds are considered potential ACR scavengers (Song et al., 2021). Following this reaction, the concentrations of both ACR and the reactive compound decrease, accompanied by the formation of new adduct products. Based on this principle, components with ACR-trapping ability typically exhibit reduced or missing chromatographic peak areas after incubation, whereas compounds lacking such reactivity generally show minimal variation in their peak responses (Hao et al., 2025; Zhuang et al., 2023). The ACR-incubation screening strategy was designed as a reaction-guided chemical approach to rapidly identify ACR-reactive constituents in *A. roxburghii* extract. The incubation condition of 37 °C in PBS buffer at pH 7.4 was selected to provide a mild aqueous environment for evaluating the intrinsic ACR-trapping capacity of extract constituents. Therefore, this condition should be interpreted as a screening condition rather than a direct simulation of thermal food processing or real food matrices. Under this condition, compounds showing marked reductions in chromatographic peak area at 254 nm after ACR incubation were considered potential ACR-reactive constituents.

In this study, the *A. roxburghii*. extract was incubated with either ACR or PBS (blank control), and the resulting mixtures were subsequently examined by UHPLC-DAD under identical analytical conditions. The chromatographic profiles indicated that all detectable constituents in *A. roxburghii*. remained unchanged after incubation with PBS (100 mM, pH 7.4) at 37 °C for 1 h (Fig. 1A), confirming their stability. To determine suitable experimental conditions for the pre-column incubation procedure, particularly the ACR concentration, the extract was reacted with different levels of ACR for 1 h. As shown in Fig. 1B, noticeable variations in the UHPLC-DAD chromatograms recorded at 254 nm were observed following treatment with 25, 50, and 100 μM ACR. 11 peaks were selected from the HPLC chromatogram of *A. roxburghii*. for analysis. The peak areas of compounds 3, 4, 5, 6, 7, 8, 10, and 11 significantly decreased or even disappeared with increasing ACR concentration, whereas the peak areas of the other compounds (1, 2, 9) showed no obvious changes in Fig. 1B. Therefore, considering the efficiency and effectiveness of the screening assay, and given that the HPLC chromatographic peak profile of *A. roxburghii*. showed pronounced changes when incubated with 100 μM ACR, the *A. roxburghii*. extract was incubated with 100 μM ACR for 1 h in this study. This condition was selected to enable comprehensive identification of potential natural ACR scavengers with strong trapping capacity. Under these conditions, 8 candidate bioactive components with ACR-scavenging activity were identified and were subsequently analyzed further.

These findings demonstrate that the proposed strategy enables rapid and efficient identification of ACR-reactive compounds from complex botanical matrices. By directly linking chemical reactivity with chromatographic variation, this approach improves the efficiency of screening natural ACR scavengers. The UHPLC-DAD-HRMS/MS platform further ensures reliable separation and structural characterization of the identified compounds.

### 3.2 Identification and quantification of ACR-scavenging constituents in *A. roxburghii* by LC-MS

In this study, the chemical constituents of *A. roxburghii*. crude extract were initially characterized using a rapid UPLC-Q-Orbitrap HRMS/MS analytical approach. The exact masses and elemental compositions of precursor and fragment ions were acquired in positive and negative ionization modes. Based on comparisons of retention times, high-resolution MS/MS fragmentation patterns, and published data and/or reference standards, a total of 35 major compounds were tentatively assigned (Xu et al., 2017). Detailed identification information is summarized in Table S2 and Fig. 2A and B, while representative total ion chromatograms are presented in Fig. S2.

**Fig. 2:**
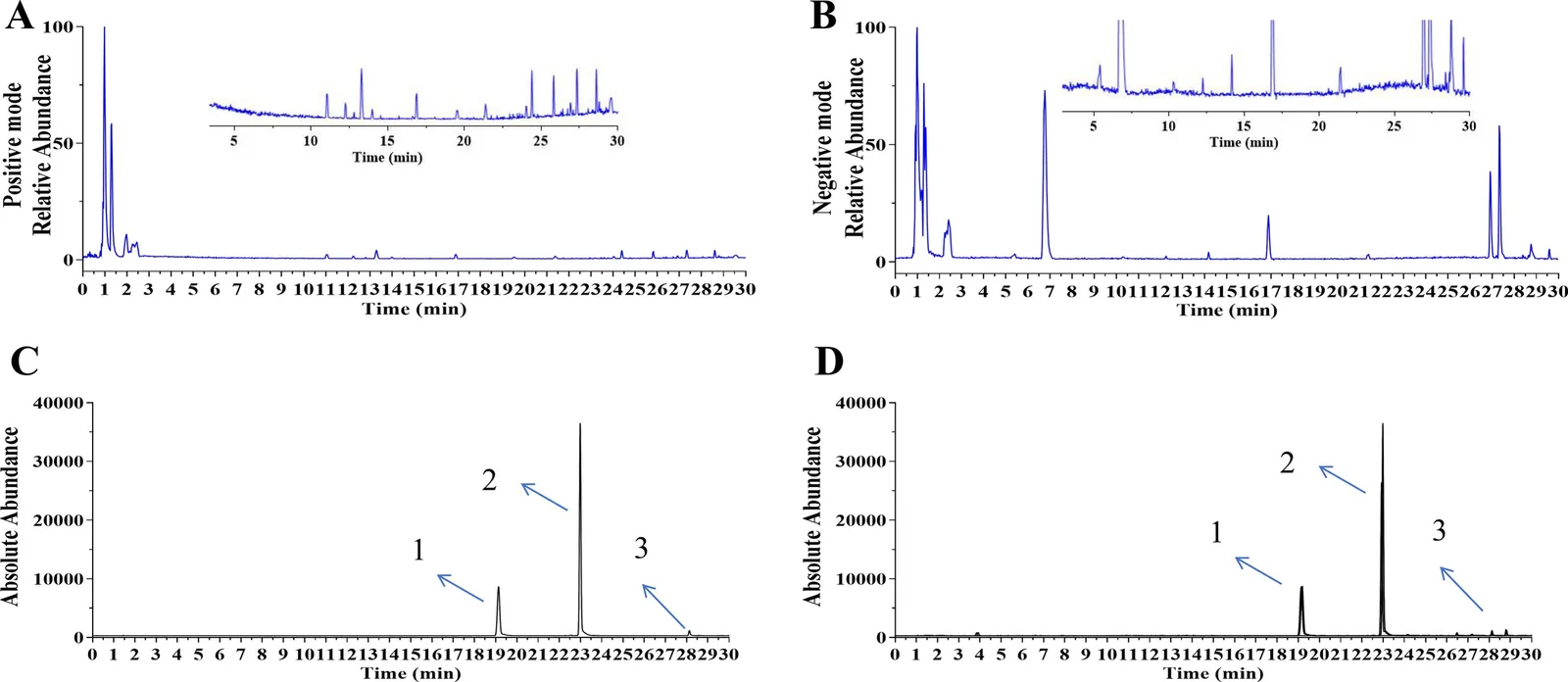
LC-MS-based identification and quantification of ACR-scavenging constituents in Anoectochilus roxburghii extract. (A and B) Representative UPLC-Q-Orbitrap HRMS/MS total ion chromatograms of *A. roxburghii* crude extract acquired in positive (A) and negative (B) ionization modes. A total of 35 major compounds were tentatively assigned based on accurate mass, MS/MS fragmentation patterns, reference standards, and literature data. (C and D) LC-QQQ-MS/MS chromatograms of mixed authentic standards (C) and *A. roxburghii* extract (D) for quantitative analysis of representative ACR-reactive compounds. (1. rutin 2. NAR, 3. ISO).

In the present study, compound 14 (tR = 14.48 min) produced an [M-H]^−^ ion at *m/z* 609.1417. The MS/MS spectrum revealed the presence of an [M-H-Glc]^−^ ion at *m/z* 193.0496 and an [M-H-Glc-H2O-COOH]^−^ ion at *m/z* 149.0603. Furthermore, the Retro-Diels-Alder feature fragment, [M-H-Glc-C3H4O3]^−^, at *m/z* 123.0447 in negative ion mode indicated that compound 14 is Rutin. Compound 23 (tR = 21.41 min) generated an [M-H]^−^ ion at *m/z* 623.1616 in the full scan mass spectrum in negative ion mode. Additionally, it exhibited [M-H-Glc]^−^ and [M-H-Glc-H2O]^−^ ions at *m/z* 225.0765 and 207.0652, respectively, leading to its identification as NAR.

Based on the screening results described in Section 3.1, the candidate compounds showing significant peak area reductions after ACR incubation were further characterized by UHPLC-DAD-HRMS/MS to determine their chemical identities and relative scavenging capacities. Eight compounds were tentatively or unequivocally identified by comparing their retention times and mass spectral data with reference standards and published literature (Xu et al., 2017). These compounds were assigned as rutin, Kaempferol 3,7-diglucoside, isoquercitrin, Quercimeritrin, Kaempferol-rutoside, NAR, ISO, and emodin. When the ACR concentration is 100 μM, the peak area reduction rates for the eight candidate compounds are 41.35 (peak 3: Rutin), 63.89 (peak 4: Kaempferol 3,7-diglucoside), 34.96 (peak 5: Isoquercitrin), 34.66 (peak 6: Quercimeritrin), 32.94 (peak 7: Kaempferol-rutoside), 42.77 (peak 8: NAR), 63.00 (peak: 10 ISO) and 49.42 (peak 11: Emodin), respectively. Among the 8 major constituents with ACR-scavenging capacity identified in the screening assay, rutin, isoquercitrin, Quercimeritrin, and Kaempferol-rutoside have been previously reported to exhibit ACR-scavenging activity (Song et al., 2021; Zhang et al., 2020; Zhu et al., 2009), supporting the accuracy and robustness of the platform.

To further clarify the relative abundance of representative ACR-reactive constituents in *A. roxburghii* extract and to support the selection of NAR for subsequent validation, three compounds, including rutin, NAR, and isorhamnetin, were quantified using LC-QQQ-MS/MS in Fig. 2C and D. As shown in Table S3, the quantitative analysis showed that rutin, NAR, and isorhamnetin were present in *A. roxburghii extract* at levels of 30.78 ± 0.28, 209.44 ± 3.81, and 2.64 ± 0.05 μg/g extract, respectively. Among the quantified compounds, NAR was the most abundant, with a content approximately 6.81-fold higher than that of rutin and 79.42-fold higher than isorhamnetin. These results indicate that NAR is a relatively abundant ACR-reactive constituent in *A. roxburghii extract*.

The selection of NAR for further evaluation was therefore based on several considerations, including its relatively high abundance, clear chromatographic and MS response, confirmed ACR reactivity in the screening assay, and limited previous investigation regarding its ACR-trapping behavior. However, although NAR was selected as a representative compound, its contribution to the overall ACR-scavenging activity of *A. roxburghii extract* should be interpreted cautiously. Several other constituents, including rutin, isorhamnetin, isoquercitrin, hirsutrin, kaempferol derivatives, and emodin, also showed ACR reactivity in the screening assay. Therefore, NAR should not be regarded as the sole active contributor to the extract. Instead, the total ACR-scavenging activity of *A. roxburghii* is likely derived from the combined effects of multiple reactive flavonoids and phenolic compounds. These findings support NAR as an abundant and representative ACR-trapping constituent, while also highlighting the multi-component nature of the ACR-scavenging activity of *A. roxburghii*.

### 3.3 *In vitro* analysis of ACR–NAR adduct formation using UPLC–HRMS/MS

To characterize the chemical interaction between NAR and ACR, reaction mixtures at molar ratios of 1:0.3, 1:1, 1:3, and 1:9 (NAR:ACR) were analyzed using UPLC-Q-Orbitrap HRMS/MS (Fig. 3). To further confirm the interaction between NAR and ACR, the reaction mixtures at the specified molar ratios were reanalyzed by UPLC-Q-Orbitrap HRMS/MS. As shown in Fig. 3A and Table 1, the total ion chromatogram (TIC) of NAR exhibited a peak at a retention time of 6.95 min. The corresponding mass spectrum showed a molecular ion at 623.1610 [M–H]^−^ (Fig. 3B), consistent with a molecular weight of 624.1690 for NAR.

**Fig. 3:**
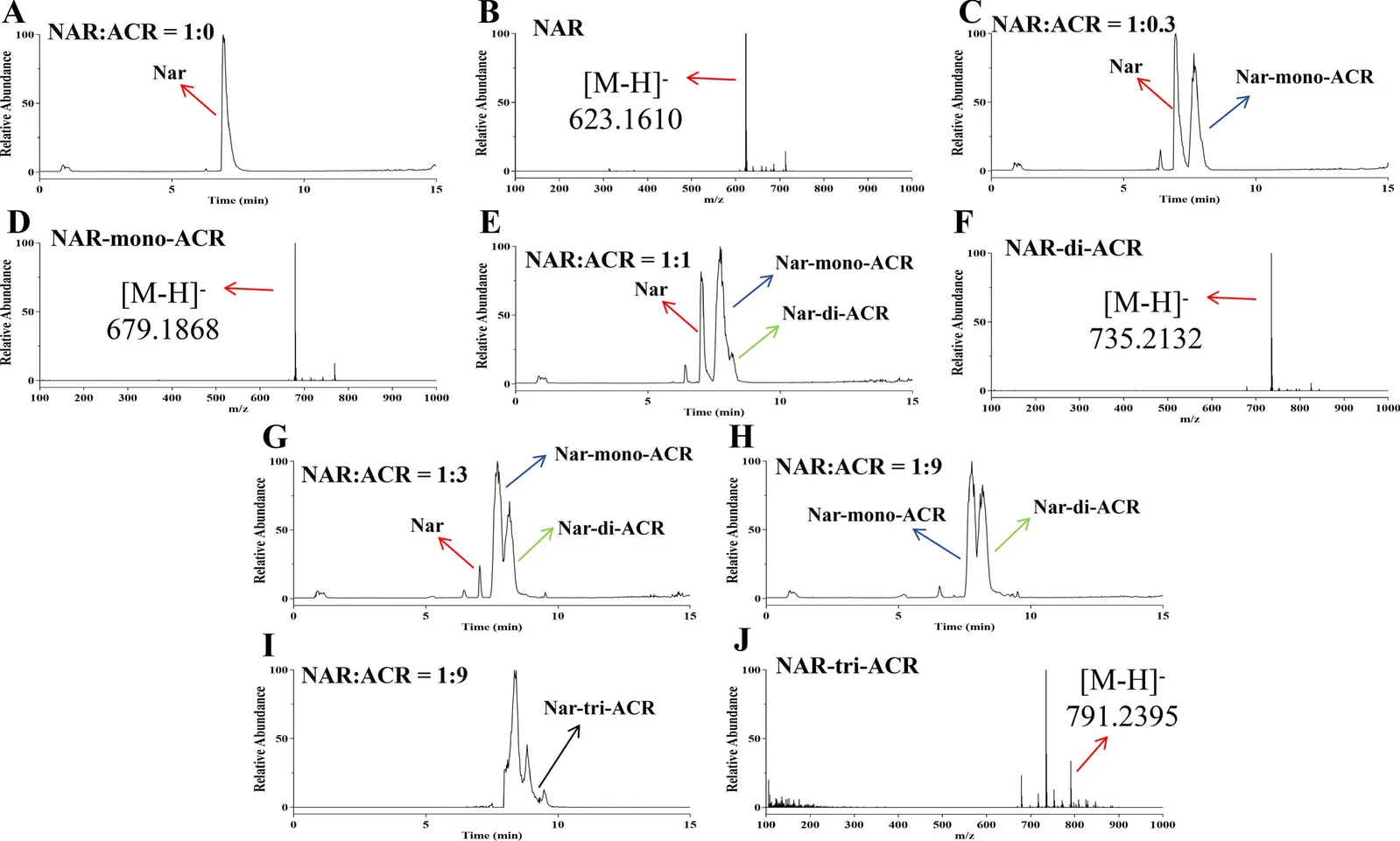
Analysis of ACR-Adduct Formation with NAR by UPLC–HR MS/MS *in vitro*. (A) TIC of NAR without ACR incubation. (B) Mass spectrum of NAR (C-H) TICs of NAR after incubation with increasing molar ratios of ACR (1:0.3, 1:1, 1:3, and 1:9), showing sequential formation of NAR-mono-ACR and NAR-di-ACR. (I) EIC of the 1 mM NAR after incubation with 9 mM of ACR. (J) Mass spectrum of NAR-tri-ACR.

**Table 1:** Typical compounds identified of the NAR-ACR adducts by UPLC-Q-Orbitrap HRMS/MS.

NO.	compound	Formula	RT (min)	Measured (***m***/***z***)	Expected m/z	Adductsion	Error (ppm)	MS/MS
1.	NAR	C_28_H_32_O_16_	7.10	623.1611	624.1690	[M-H]^−^	0.656	315.0507, 300.0269, 271.0256
2.	NAR-mono-ACR	C_31_H_36_O_17_	7.77	679.1868	680.1952	[M-H]^−^	−0.068	623.1626, 371.0762,
3.	NAR-di-ACR	C_34_H_40_O_18_	8.18	735.2132	736.2215	[M-H]^−^	0.149	679.1874, 427.1030, 371.0464
4.	NAR-tri-ACR	C_37_H_44_O_19_	8.84	791.2133	792.2477	[M-H]^−^	0.019	735.2142, 679.1866, 483.1279, 427.1018,

After 1 h of incubation of NAR with ACR (1:0.3 ratio, NAR:ACR), a new peak assigned as NAR-mono-ACR appeared at RT = 15.8 min (Fig. 3C). In the mass spectrum (Fig. 3D), this peak exhibited a [M-H]^−^ ion at *m/z* 679.1868, which is 56 amu higher than that of free NAR (*m/z* 623.1610), indicating the formation of a mono-adduct with ACR (Mw 56). The relative abundance of the NAR-mono-ACR adduct increased when the molar ratio of ACR to NAR was 1:1 (Fig. 2E), and the unreacted NAR levels were significantly reduced. In addition, at a retention time of 8.18 min (Fig. 3E), the product with *m/z* of 735.2132 [M-H]^−^ was detected in Fig. 3F, which was 112 mass units larger than the NAR, indicating the NAR formated of a NAR-di-ACR adduct with two molecules of ACR. As the ACR ratio increases (Fig. 3G and H), the abundance of NAR gradually decreases, being replaced by single ACR adducts and double ACR adducts. At an NAR:ACR molar ratio of 1:9, an additional ion at *m/z* 735.2134 was observed in extracted ion chromatograms at RT = 8.84 min, corresponding to a mass increase of 168 Da relative to NAR, which was assigned to a NAR-tri-ACR adduct, as shown in Fig. 3I and J. Detailed identification information is summarized in Fig. S2. These results demonstrate that NAR can react directly with ACR to form multiple adducts, and the formation of these conjugates is strongly dependent on the concentration of ACR in the reaction system.

In the NAR–ACR co-incubation experiment, increasing concentrations of ACR were used to clarify the ACR-trapping behavior of NAR and to explore the possible number of reactive sites involved in adduct formation. The ACR concentration range of 0.3–9 mM was selected based on the molar ratio between NAR and ACR, rather than on physiological or dietary exposure levels. Therefore, the highest ACR concentration used in this assay should not be interpreted as a realistic exposure concentration *in vivo* or in food systems. The purpose of using excess ACR was to drive the chemical reaction sufficiently and to facilitate the detection of different NAR–ACR adducts by UPLC-HRMS/MS. Under these conditions, mono-, di-, and tri-ACR adducts were detected, suggesting that NAR possesses multiple potential ACR-reactive sites. Since this was a cell-free chemical reaction system, the potential cytotoxicity or animal toxicity caused by high ACR levels was not involved. Thus, the concentration gradient was used mainly for adduct enrichment and structural characterization.

### 3.4 Analysis of the formation of ACR adducts of NAR *in vivo* by UPLC-HR MS/MS

The data presented in Section 3.3 demonstrate that NAR is capable of capturing ACR and forming adducts under *in vitro* conditions. This prompted us to investigate whether a similar pathway operates *in vivo*, whereby NAR might also scavenge ACR through adduct formation (Jia, Gu, et al., 2023; Lu et al., 2021). To evaluate this, mice were orally administered NAR (100 mg/kg body weight), and fecal samples were collected over 24 h for metabolite analysis. The methodology of the experiment is depicted in Fig. 4A. UPLC–MS/MS was used to detect NAR, its metabolites, and their potential ACR adducts.

**Fig. 4:**
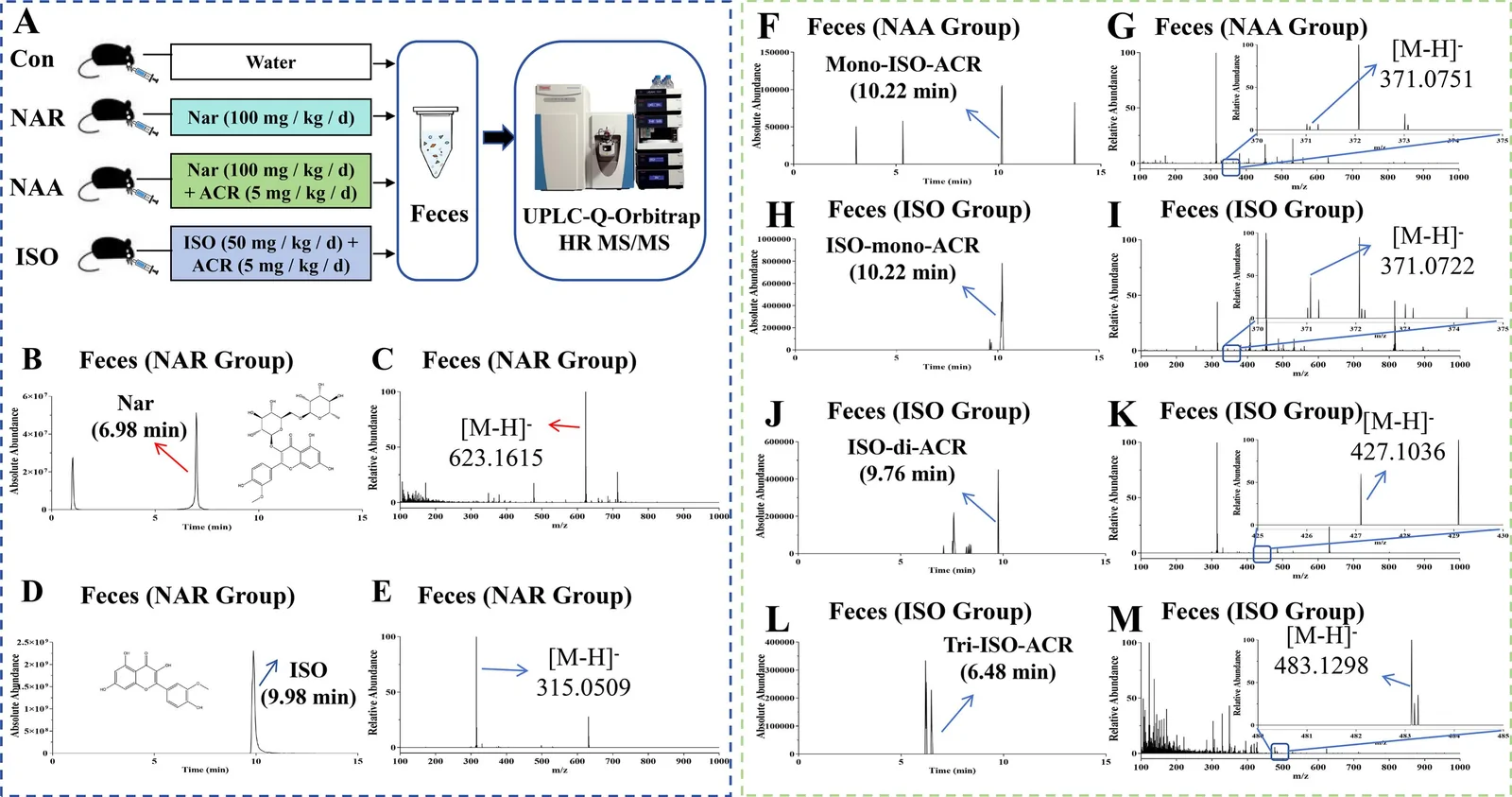
Analysis of ACR-Adduct formation with NAR by UPLC-HR MS/MS *in vivo*. (A) Experimental design. (B–E) EICs and corresponding high-resolution mass spectra spectra of the standard of NAR and its metabolism (ISO) in NAR group mouse fecal samples. Identification of ISO-ACR adducts in mouse fecal samples. (F–M) EICs and corresponding high-resolution mass spectra showing the formation of ISO-mono-ACR adducts in fecal samples from the NAA group (F and G) and ISO group (H and I), and ISO-di-ACR (J and K) and ISO-tri-ACR (L and M) adducts in fecal samples from the ISO group.

As shown in Fig. 4B, a chromatographic peak detected at RT = 7.03 min with a parent ion at *m*/*z* 623.1615 [M–H]^−^ was identified as NAR based on its retention behavior and MS^2^ fragmentation pattern compared with the authentic standard (Fig. 4C). In addition, ISO, a major metabolite of NAR, was detected at RT = 9.88 min with an [M–H]^−^ ion at *m*/*z* 315.0509 (Fig. 4D and E), indicating that NAR undergoes biotransformation *in vivo*. Consistently, extracted ion chromatograms further confirmed the presence of both NAR and ISO, although the signal intensity of ISO was markedly higher, suggesting extensive metabolic conversion of NAR to ISO in NAR + ACR group (NAA) group (Fig. S3). It has been reported that hesperidin can undergo enzymatic hydrolysis by intestinal microbiota, where glucosidases cleave the rutinose group, allowing the resulting aglycone hesperidin to be absorbed (Jia, Gu, et al., 2023). Similarly, it is reasonable to speculate that NAR may also be metabolized *in vivo* into its corresponding aglycone (ISO), which can subsequently be absorbed. NAR is a flavonoid glycoside, and its conversion may involve deglycosylation reactions mediated by gut microbiota-derived glycosidases and/or host metabolic enzymes. Since fecal microbiota incubation, antibiotic intervention, hepatic microsomal incubation, or enzyme-specific inhibition experiments were not performed in the present study, it is not possible to determine whether intestinal microbiota or hepatic metabolism plays the dominant role in this conversion. Therefore, the current results support the occurrence of NAR-to-ISO conversion *in vivo*, but the underlying metabolic pathway requires further investigation. To further clarify the *in vivo* role of NAR in ACR scavenging, an additional ISO-treated group was included to evaluate the contribution of this metabolite to ACR trapping.

Importantly, no NAR–ACR adduct was detected in the fecal samples. Instead, the predominant conjugate observed was the ISO–ACR adduct in mice treated with either NAR or ISO (Fig. 4 and Table S4). Furthermore, ACR adducts of ISO in fecal samples were identified using extracted ion chromatogram analysis (Fig. 4). A chromatographic peak corresponding to the ISO-mono-ACR adduct was detected at 10.22 min by extracting the parent ion at *m*/*z* 371.07 [M–H]^−^ in both the NAA and ISO groups (Fig. 4F, G, H and I). Likewise, extraction of the ion at *m*/*z* 427.10 [M–H]^−^ revealed a peak at 9.76 min, which was assigned to the ISO-di-ACR adduct in the ISO group (Fig. 4J and K). Additionally, a peak observed at 6.48 min under the ion channel *m*/*z* 483.12 [M–H]^−^ was attributed to the ISO-tri-ACR adduct (Fig. 4L and M). Detailed identification information is summarized in Table S4. This finding indicates that NAR may exert its ACR-scavenging effect *in vivo* primarily through its metabolite ISO rather than in its parent form. The formation of the ISO–ACR adduct may result either from direct trapping of ACR by ISO generated from NAR metabolism or from structural conversion of initially formed NAR–ACR conjugates, although the latter possibility requires further investigation. These results provide *in vivo* evidence that NAR can indirectly contribute to ACR detoxification through its metabolite ISO, supporting its potential role as a natural ACR scavenger.

Importantly, this finding raises the possibility that ISO could represent a more direct and efficient ACR scavenger for future applications. Nevertheless, NAR may function as a precursor compound that can be converted into the active scavenger after ingestion, thereby contributing indirectly to ACR detoxification. Such precursor–metabolite relationships may offer advantages in terms of compound stability, bioavailability, and sustained *in vivo* activity. From a practical standpoint, both NAR and ISO may have potential as functional food ingredients for reducing ACR exposure. Therefore, both NAR and ISO merit further investigation as functional ACR scavengers. Comparative studies evaluating their bioavailability, metabolic stability, and *in vivo* detoxification efficiency are warranted to determine the optimal candidate for future food and nutraceutical applications aimed at reducing ACR-related health risks.

The 5 mg/kg ACR gavage model was designed as a short-term *in vivo* ACR-trapping validation experiment rather than a complete *in vivo* efficacy study. The main purpose of this experiment was to determine whether NAR or its metabolite ISO could react with ACR *in vivo* and generate detectable ACR adducts in feces. Therefore, this experiment mainly provided chemical evidence for gastrointestinal ACR trapping, which was consistent with the *in vitro* formation of NAR–ACR adducts.

Nevertheless, the present study did not directly evaluate the stability of NAR or its ACR-scavenging efficiency under realistic food processing conditions. The *in vitro/vivo* ACR incubation system used here was designed to confirm the chemical reactivity of NAR toward ACR and to characterize NAR–ACR adduct formation, rather than to reproduce actual food-processing environments. Therefore, the application of NAR in food systems should be interpreted cautiously. Further studies are needed to determine its thermal stability, pH stability, and ACR-scavenging performance in simulated and real food matrices, including acidic beverages, lipid-rich heated systems, baked foods, and ultra-processed foods. Such studies will be essential to evaluate whether NAR can function as an effective food-compatible carbonyl scavenger under practical processing and storage conditions.

### 3.5 Effect of NAR on the formation of AGEs

Since ACR is a highly reactive carbonyl compound capable of accelerating AGEs formation. To more comprehensively evaluate the antiglycation-related activity of NAR, MGO-BSA, Glu-BSA, and Fru-BSA models were also included. The inhibitory effects observed in these models suggest that NAR may modulate protein modification under both reactive carbonyl- and reducing sugar-driven conditions. Therefore, the ACR-BSA system used in this study should be interpreted as a model of ACR-induced protein carbonyl/glycoxidative modification rather than a classical glycation model. In contrast, the MGO-BSA, Glu-BSA, and Fru-BSA systems represent glycation-related models driven by reactive dicarbonyls or reducing sugars.

The inhibitory effect of NAR on AGEs generation was evaluated using four *in vitro* glycation models (ACR–BSA, MGO–BSA, Glu–BSA, and Fru–BSA) (Wang et al., 2019). AGEs formation was monitored through characteristic autofluorescence, a well-established analytical approach for tracking glycation progression (Liu et al., 2022). Glycation is a non-enzymatic reaction between reducing sugars or reactive carbonyl species and free amino groups of proteins, leading to the formation of AGEs, which progressively accumulate and contribute to cellular dysfunction (Kathuria et al., 2023). AGEs are irreversibly generated *in vivo*, progressively accumulate, and have the potential to disrupt cellular function (Zgutka et al., 2023). The formation of AGEs can be reduced by applying specific inhibitors, including various natural substances and synthetic agents. As shown in Fig. 5, NAR exhibited a significant inhibitory impact on fluorescent AGEs at 7 days in these model systems. In the 4 models, NAR inhibited AGEs in a dose-dependent manner. In parallel, AG, a commonly used positive control in previous studies, was selected to compare the AGEs-inhibitory activity of NAR. The results showed that both NAR and the positive control AG significantly inhibited AGEs formation in all glycation systems. The IC₅₀ values of NAR were 57.44 μM (ACR-BSA, Fig. 5A), 86.32 μM (MGO-BSA, Fig. 5B), 61.30 μM (Glu-BSA, Fig. 5C), and 62.73 μM (Fru-BSA, Fig. 5D), respectively, demonstrating its potent inhibitory activity. The inhibition rate was plotted against the logarithm of NAR concentration, and IC50 was defined as the concentration of NAR required to inhibit 50% of fluorescence formation. As the four models used different inducers and concentrations, the IC₅₀ values obtained from these systems should be interpreted within the context of each individual model. Direct comparison of IC₅₀ values among the ACR–BSA, MGO–BSA, Glu–BSA, and Fru–BSA models is not sufficient to determine whether NAR preferentially inhibits one pathway over another. Therefore, the inhibitory effect observed in the ACR–BSA model is better interpreted as being consistent with the direct ACR-trapping capacity of NAR, as supported by the formation of NAR–ACR adducts, rather than as definitive evidence that NAR is more active in the ACR-induced model than in other glycation systems. Notably, the strongest inhibition observed in the ACR-induced model further supports the ability of NAR to trap reactive carbonyl species such as ACR.

**Fig. 5:**
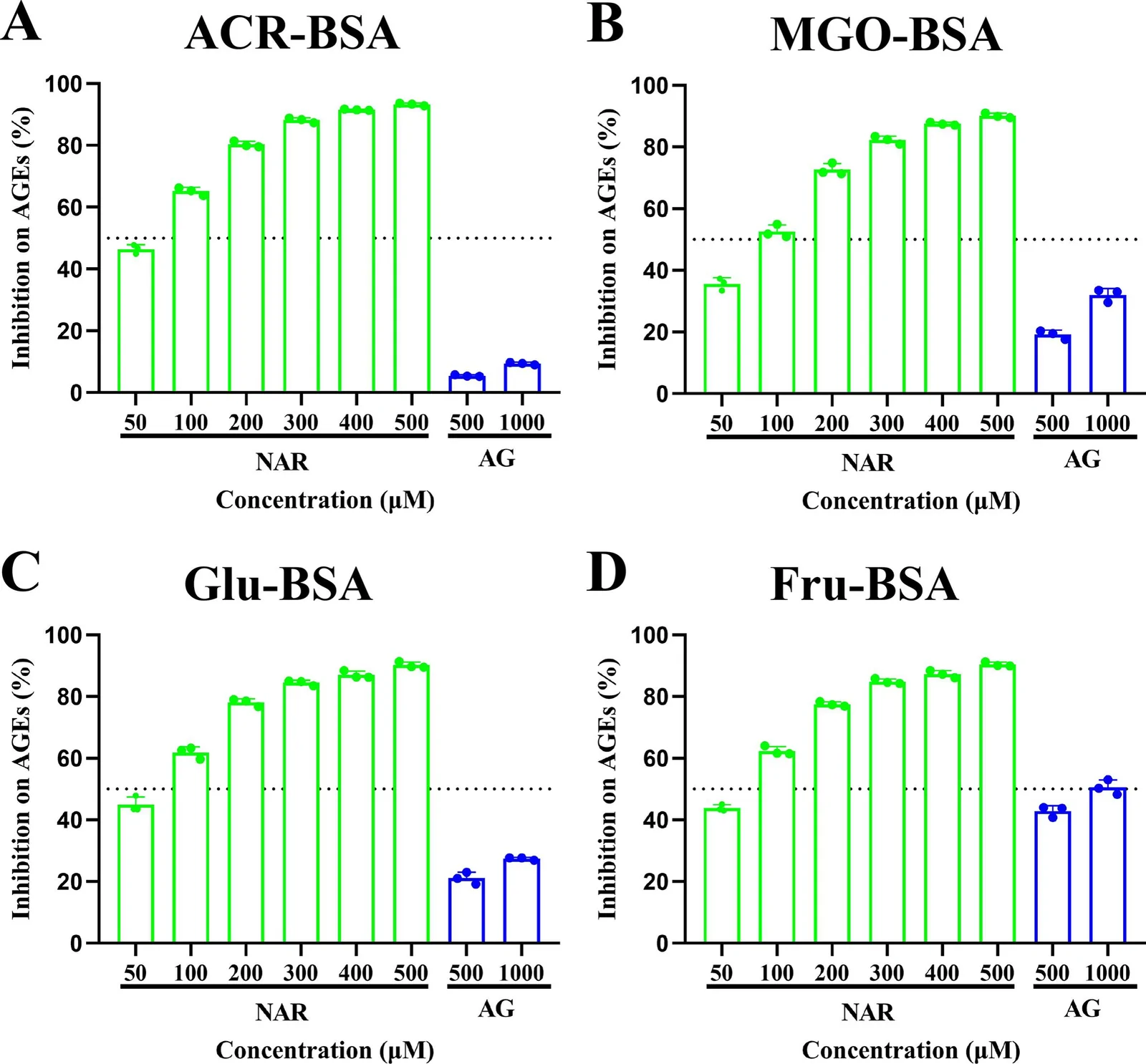
*In vitro* inhibitory effects of NAR on AGEs formation and protein glycoxidation. (A-D) Inhibition of AGEs by different concentrations of NAR and phloretin for 7 days in ACR-BSA model (A), MGO-BSA model (B), Glu-BSA model (C) and Fru-BSA model (D). Amino guanidine (AG) was employed as the positive control in anti-glycation.

In the antiglycation assays, AG was used as a classical positive control for AGE formation inhibition. Compared with AG, NAR exhibited comparable inhibitory activity, highlighting its potential as a natural alternative for controlling AGEs formation. These findings indicate that the carbonyl-trapping ability of NAR may play a central role in suppressing AGEs formation and provide mechanistic support for its protective effects against carbonyl-stress-related damage. Importantly, since AGEs accumulation is closely linked to protein aggregation and neurodegenerative processes (Bhattacharya et al., 2023), the observed inhibition of AGEs formation by NAR may contribute to its protective effects against amyloid aggregation (Anwar et al., 2021; Gostomska-Pampuch et al., 2022).

ACR was selected as the principal screening target in this study because it is an important reactive carbonyl species generated during lipid peroxidation and thermal food processing. Unlike classical reactive dicarbonyl AGE precursors such as MGO and GO, ACR is more closely associated with protein carbonylation, acrolein-derived adduct formation, and carbonyl stress-related protein modification. Therefore, the ACR-incubation UHPLC-DAD-HRMS/MS strategy was primarily designed to identify natural constituents with ACR-trapping capacity from *A. roxburghii*, rather than to screen inhibitors specific to MGO- or GO-derived AGE formation. The formation of NAR-ACR adducts confirmed that NAR could directly trap ACR, providing chemical evidence for its ability to attenuate ACR-related carbonyl stress. However, this adduct-forming behavior should not be interpreted as the sole explanation for the inhibitory effect of NAR on AGE formation. MGO and GO are well-recognized precursors of major AGEs such as MG-H1, CEL, G-H1, and CML, whereas ACR mainly contributes to acrolein-derived protein modifications and ALE-type carbonyl stress. Therefore, the inhibitory effect observed in the ACR–BSA system is more appropriately described as suppression of ACR-induced protein carbonyl/glycoxidative modification.

### 3.6 Effects of NAR on protein glycoxidation products and radical-scavenging activity

Protein glycoxidation, involving the combined action of non-enzymatic glycation and oxidation, plays a critical role in AGEs formation (Jia, Guo, et al., 2023). During this process, amino acid residues such as tryptophan and tyrosine are particularly susceptible to modification, resulting in the generation of characteristic fluorescent products, including dityrosine and N-formylkynurenine, which serve as important biomarkers for monitoring protein oxidation and AGEs progression (Peng et al., 2008). Specifically, tryptophan residues can be converted into dityrosine and N-formyl kynurenine, while tyrosine residues can undergo transformation into kynurenine (Wu et al., 2019). NAR exhibited substantial, dose-dependent inhibition of these protein glycoxidation products across all concentration levels. At a concentration of 200 μM, NAR exhibited strong inhibitory effects on oxidative protein modification in the ACR–BSA model, with inhibition rates of 80.66%, 83.09%, and 75.13% for N-formylkynurenine (Fig. 6A), dityrosine (Fig. 6B), and kynurenine (Fig. 6C), respectively. Similarly, in the MGO–BSA system (Fig. S5A), the inhibition rates of NAR against the formation of N-formylkynurenine, dityrosine, and kynurenine were 73.33%, 78.73%, and 52.38%, respectively. In the Glu–BSA model (Fig. S5B), the corresponding inhibition rates were 78.70%, 78.34%, and 75.38%, respectively. Likewise, in the Fru–BSA system (Fig. S5C), NAR suppressed the formation of these oxidation products, with inhibition percentages of 77.83%, 77.50%, and 74.63%, respectively. These findings demonstrate that NAR effectively suppresses protein glycoxidation during the glycation process, thereby inhibiting oxidative modifications associated with the formation of AGEs.

**Fig. 6:**
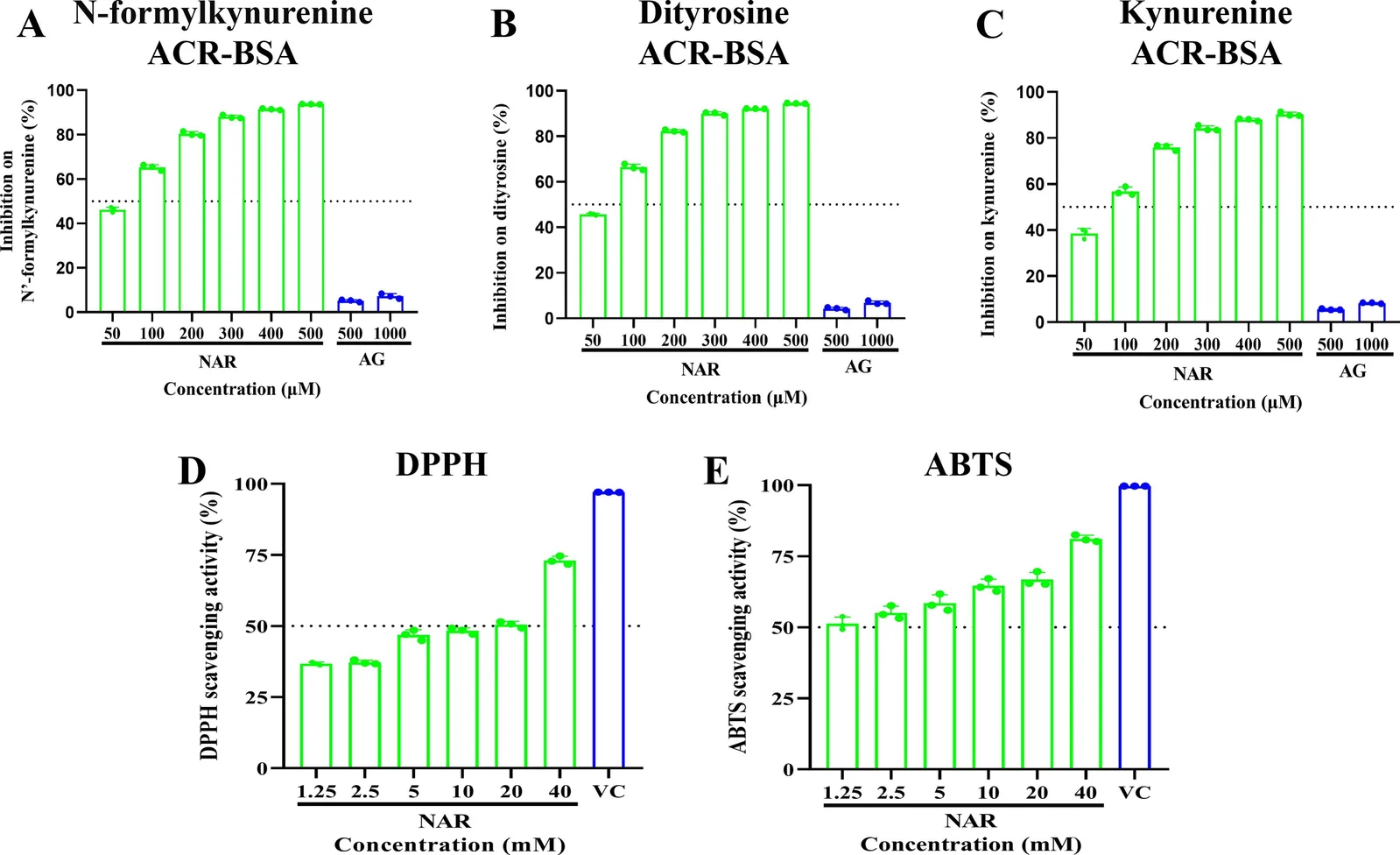
*In vitro* inhibitory effects of NAR on protein glycoxidation. (A-C) Effects of three kinds of NAR on the formation of N-formylkynurenine (A), dityrosine (B) and kynurenine (C) in ACR-BSA model. AG was employed as the positive control in anti-glycation. Antioxidant activity effect of NAR. (D) The DPPH radical-scavenging activity of NAR. (E) The ABTS radical-scavenging activity of NAR. Vitamin C (VC) (1.25 mM) was employed as the positive control in antioxidant activity.

It should be noted that the ACR–BSA, MGO–BSA, Glu–BSA, and Fru–BSA systems were established using different inducers and concentrations. Therefore, the inhibition rates among these models should not be directly compared as evidence that NAR is more effective in one system than another. The inhibitory effect observed in the ACR-BSA model is better interpreted as being consistent with the ACR-trapping capacity of NAR, as supported by NAR-ACR adduct formation, rather than as definitive evidence of stronger activity in the ACR-induced model. Since ACR primarily induces protein carbonylation and oxidative protein modification, the ACR–BSA system should be regarded as an ACR-induced protein carbonyl/glycoxidative modification model rather than a classical glycation model. By trapping reactive carbonyl intermediates and suppressing the formation of oxidative biomarkers such as N-formylkynurenine, dityrosine, and kynurenine, NAR can interrupt the glycation–oxidation cascade and consequently limit the generation of irreversible AGEs. This protective effect against glycoxidation is particularly important, as oxidative modification of proteins is closely associated with structural destabilization and aggregation (Park et al., 2020).

The relationship between glycation and oxidative stress has been widely recognized. Oxidative stress arises from an imbalance between the production of reactive oxygen species (ROS) and the antioxidant defense capacity in biological systems. Under these conditions, elevated ROS levels promote oxidative reactions that accelerate the formation and accumulation of AGEs (Gao et al., 2024). Therefore, evaluating the antioxidant activity of NAR represents a critical aspect of this study. The antioxidant capacity of NAR was evaluated using the complementary DPPH and ABTS radical scavenging assays, which are widely applied to assess the free radical scavenging ability of natural antioxidants. Antioxidant activity was expressed as the IC₅₀ value, defined as the concentration required to reduce the initial DPPH or ABTS radical level by 50%, reflecting the radical scavenging efficiency (L. Wu et al., 2021). The results demonstrated that NAR exhibited potent antioxidant activity, with IC50 values of 8.23 mM in the DPPH assay and 1.39 mM in the ABTS assay in Fig. 6D and E. Compared with vitamin C, NAR exhibited weaker radical-scavenging activity; therefore, its antioxidant capacity should be described as moderate. This result suggests that the inhibitory effect of NAR on AGE/carbonyl stress-related protein modification is unlikely to be mainly dependent on direct free radical scavenging. The results indicated that NAR can reduce or neutralize the formation of free radicals, suggesting that it may lower the levels of radical species generated during the formation of AGEs. Natural products may inhibit AGE formation and glycoxidative protein modification through multiple mechanisms, including reactive carbonyl trapping, suppression of dicarbonyl intermediates, protection of protein amino groups, metal chelation, inhibition of oxidative propagation, and attenuation of protein oxidation. In the present study, the formation of NAR–ACR adducts provides direct evidence for the carbonyl-trapping capacity of NAR. Therefore, NAR may suppress ACR-induced protein carbonyl/glycoxidative modification mainly through reactive carbonyl scavenging, while its moderate radical-scavenging activity may provide auxiliary protection by reducing oxidative propagation during glycoxidation. These complementary mechanisms may jointly contribute to the inhibitory effects of NAR on protein modification and carbonyl stress-associated damage.

### 3.7 NAR and ISO attenuated serum carbonyl stress, oxidative stress, and inflammation in low-dose ACR-exposed mice

To further evaluate the biological effects of NAR and ISO under repeated low-dose ACR exposure, mice were orally administered ACR at 3.5 mg/kg body weight for 14 consecutive days. As shown in Fig. S4, ACR exposure significantly reduced body weight compared with the control group (Fig. S4A), while increasing the liver and spleen indexes (Fig. S4 B and Fig. S4 D). No significant difference was observed in the kidney index (Fig. S4C). These results indicate that repeated low-dose ACR exposure induced measurable systemic effects, particularly involving changes in liver and spleen indexes. Treatment with NAR and ISO partially reversed these alterations, as reflected by improved body weight and reduced liver and spleen indexes, suggesting that both compounds alleviated ACR-induced physiological disturbances.

To further investigate the effects of NAR and ISO on ACR-induced serum biochemical responses, serum glycation/carbonyl stress, oxidative stress, and inflammatory markers were determined. AGEs and CML were measured as representative glycation/carbonyl stress-related markers; MDA was used as an indicator of lipid peroxidation, whereas SOD activity was measured to evaluate antioxidant defense capacity. IL-1β, IL-6, and TNF-α were determined as representative inflammatory cytokines. As shown in Fig. 7, repeated ACR exposure significantly increased serum AGEs and CML levels compared with the control group, indicating that ACR induced obvious serum carbonyl/glycation stress (Fig. 7A and B). Meanwhile, ACR exposure markedly decreased SOD activity and increased MDA levels, suggesting impaired antioxidant defense and enhanced lipid peroxidation in Fig. 7C and D. In addition, serum IL-1β, IL-6, and TNF-α levels were significantly elevated in the ACR model group, indicating that repeated low-dose ACR exposure triggered a systemic inflammatory response (Fig. 7E–G). Notably, treatment with NAR and ISO significantly attenuated these ACR-induced abnormalities. Both low- and high-dose NAR and ISO reduced serum AGEs and CML levels, restored SOD activity, decreased MDA accumulation, and suppressed the elevation of IL-1β, IL-6, and TNF-α. These findings suggest that NAR and ISO can alleviate ACR-induced serum carbonyl/glycation stress, oxidative stress, and inflammatory responses *in vivo*. Together with the *in vitro* ACR-trapping results, these data further support the potential of NAR and its metabolite ISO to mitigate ACR-associated biological disturbances through carbonyl scavenging and partial regulation of oxidative and inflammatory responses.

**Fig. 7:**
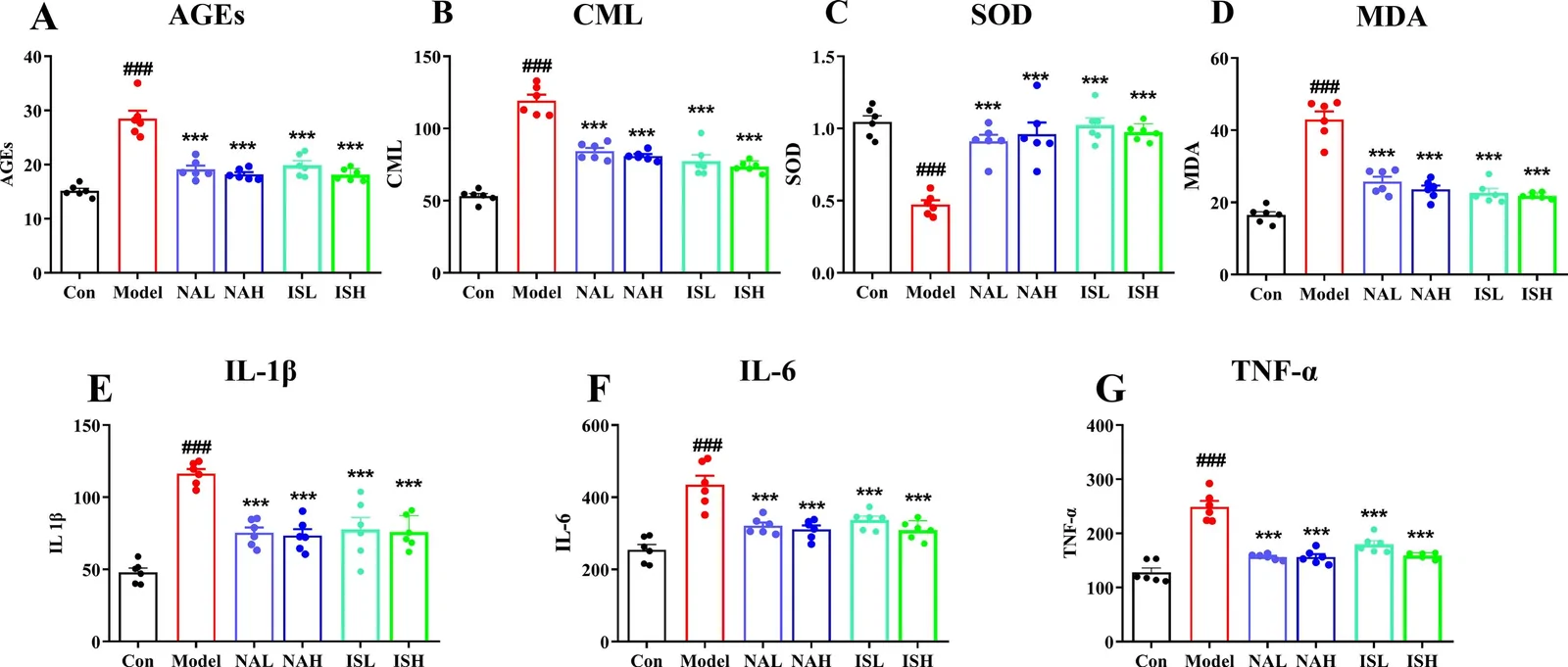
Effects of NAR and ISO on serum glycation/carbonyl stress, oxidative stress, and inflammatory markers in low-dose ACR-exposed mice. (A) AGEs. (B) CML. (C) SOD. (D) MDA. (E) IL-1β. (F) IL-6. (G) TNF-α. Con, control group; Model, ACR model group; NAL, ACR + low-dose NAR group; NAH, ACR + high-dose NAR group; ISL, ACR + low-dose ISO group; ISH, ACR + high-dose ISO group. The data are expressed as the means ± SEM (*n* = 6). (^#^p<0.05, ^##^p<0.01 and ^###^p<0.001 *vs.* Con; ^⁎^p<0.05, ^⁎⁎^p<0.01 and ^⁎⁎⁎^p<0.01 *vs.* Model)

## 4 Conclusion

In this study, a reaction-guided UHPLC-DAD-HRMS/MS strategy was developed for the rapid identification of ACR-scavenging constituents from Anoectochilus roxburghii. Eight major ACR-reactive compounds were identified, among which NAR was selected as a representative compound due to its relatively high abundance, clear MS response, and confirmed ACR reactivity.

NAR showed direct ACR-trapping capacity *in vitro* through the formation of mono-, di-, and tri-ACR adducts. *In vivo* fecal analysis further suggested that NAR could be converted into ISO, which contributed to ACR trapping by forming ISO–ACR adducts. In addition, NAR inhibited protein modification in ACR–BSA, MGO–BSA, Glu–BSA, and Fru–BSA models and reduced protein glycoxidation products, suggesting its potential to attenuate carbonyl stress-related protein damage.

The low-dose subacute ACR exposure experiment further showed that NAR and ISO alleviated ACR-induced physiological and serum biochemical abnormalities, including increased AGEs, CML, MDA, IL-1β, IL-6, and TNF-α levels and decreased SOD activity. Overall, these findings identify NAR as a representative ACR-trapping constituent of *A. roxburghii* and support its potential role in mitigating ACR-induced carbonyl stress, oxidative stress, and inflammation. Further studies are needed to evaluate its stability and scavenging efficiency in realistic food-processing systems and to clarify its interactions with major AGE precursors and acrolein-derived ALEs.

## CRediT authorship contribution statement

**Guo Dong Zhuang:** Writing – review & editing, Writing – original draft, Methodology, Formal analysis, Data curation, Conceptualization. **Yong Pei Tang:** Methodology, Data curation. **Rui Long Zhang:** Data curation, Conceptualization. **Hang Hong Lo:** Data curation, Conceptualization. **Lin Lin Song:** Methodology, Data curation, Conceptualization. **Xiao Yun Yun:** Methodology, Conceptualization. **Jian Hui Wu:** Data curation, Conceptualization. **Cai Ren Wang:** Data curation, Conceptualization. **Hui Miao Wang:** Data curation, Conceptualization. **Meng Han Liu:** Data curation, Conceptualization. **Zhi Kang Wang:** Methodology. **Vincent Kam Wai Wong:** Methodology, Data curation. **Betty Yuen Kwan Law:** Writing – review & editing, Writing – original draft, Funding acquisition.

## Declaration of competing interest

The authors declare that they have no known competing financial interests or personal relationships that could have appeared to influence the work reported in this paper.

## Data Availability

Data will be made available on request.
