# Supplementary material for: Rapid identification of acrolein scavengers from Anoectochilus roxburghii and evaluation of their representative antiglycation and antioxidant activities
Source: Food Chem X. 2026 Oct 7;39:104585. doi: 10.1016/j.fochx.2026.104585 (PMC13677956; doi:10.1016/j.fochx.2026.104585)
Supplement: Supplementary file 1 [file mmc1.docx]

**Supporting Information**

**Rapid identification of acrolein scavengers from Anoectochilus roxburghii and evaluation of their representative antiglycation and antioxidant activities**

Guo Dong Zhuang ^a^, Yong Pei Tang ^a^, Rui Long Zhang ^a^, Hang Hong Lo ^a^, Lin Lin Song ^a^, Xiao Yun Yun ^a^, Jian Hui Wu ^a^, Cai Ren Wang ^a^, Hui Miao Wang ^a^, Meng Han Liu ^a^, Zhi Kang Wang ^a^, Vincent Kam Wai Wong ^a, b^, Betty Yuen Kwan Law ***** ^a, b^

**Affiliations**

*^a^ Dr. Neher’s Biophysics Laboratory for Innovative Drug Discovery, The State Key Laboratory of Mechanism and Quality of Chinese Medicine, Faculty of Chinese Medicine, Macau University of Science and Technology, Macau, China*

*^b^ Macau University of Science and Technology Zhuhai MUST Science and Technology Research Institute, China*

****Corresponding authors***

Betty Yuen Kwan Law: Tel: (853) 88972407; Fax: (853) 28822799; E-mail: yklaw@must.edu.mo

**Fig. S1.** The structures of the compounds identified in A. roxburghii.

**Fig. S2** Formation and proposed structures of NAR and its ACR adducts.

**Fig. S3** Analysis of ACR-Adduct Formation with NAR by UPLC-HR MS/MS in vivo. (A) Experimental design. (B-E) Chromatogram and ESI-MS^2^ spectra of the standard of NAR and its ISO in NAA group mouse Feces.

**Fig. S4** Effects of four kinds of NAR and AG on the formation of N’-formylkynurenine, dityrosine and kynurenine. BSA-MGO model (A), BSA-Glc model (B) and BSA-Fru model (C).

**Fig. S5** Effects of NAR and ISO on body weight and organ indexes in low-dose ACR-exposed mice.

(A) Body weight. (B) Liver index. (C) Kidney index. (D) Spleen index. Con, control group; Model, ACR model group; NAL, ACR + low-dose NAR group; NAH, ACR + high-dose NAR group; ISL, ACR + low-dose ISO group; ISH, ACR + high-dose ISO group. The data are expressed as the means ± SEM (n = 6). (^#^p＜0.05, ^##^p＜0.01 and ^###^p＜0.001 vs. Con; ^*^p＜0.05, ^**^p＜0.01 and ^***^p＜0.01 vs. Mod.)

**Table S1** MRM transitions and calibration parameters for the quantification of representative ACR-reactive compounds in A. roxburghii extract by LC-QQQ-MS/MS

| **NO.** | **compound** | **RT (min)** | **Ion mode** | **Precursor ion**  ***(m/z)*** | **Product ion**  ***(m/z)*** | **Fragmentor**  **(V)** | **CE**  **(eV)** |
| --- | --- | --- | --- | --- | --- | --- | --- |
| 1. | Rutin | 19.140 | ESI^-^ | 609.1 | 299.9 | 135 | 40 |
| 2. | NAR | 22.987 | ESI^-^ | 623.5 | 315.8 | 225 | 20 |
| 3. | ISO | 28.133 | ESI^-^ | 315.4 | 300.1 | 150 | 30 |

RT, retention time; CE, collision energy. The listed MRM transitions should be confirmed and optimized using authentic standards under the final LC-QQQ-MS/MS conditions.

**Table. S2.** Typical compounds identified from A. roxburghii. by HPLC-Q-Orbitrap HRMS/MS.

| **NO.** | **compound** | **Formula** | **RT (min)** | **Measured**  **(m/z)** | **Expected**  **m/z** | **Adductsion** | **Error**  **(ppm)** | **MS/MS** |
| --- | --- | --- | --- | --- | --- | --- | --- | --- |
| 1. | Hexose | C_6_H_11_O_6_ | 0.97 | 179.0551 | 180.0634 | [M-H]^-^ | 0.533 | NA |
| 2. | Gallic acid | C_7_H_6_O_5_ | 1.60 | 169.0134 | 170.0215 | [M-H]^-^ | 1.185 | 125.0230, 107.0236 |
| 3. | Adenosine | C_10_H_13_N_5_O_4_ | 1.99 | 266.1039 | 267.0968 | [M+H]^+^ | -0.561 | 136.0616, 119.0652 |
| 4. | Thymidine | C_10_H_13_N_2_O_5_ | 4.12 | 241.0827 | 242.0903 | [M-H]^-^ | 3.285 | 125.0231 |
| 5. | D-pantothenic acid | C_9_H_17_NO_5_ | 5.48 | 2811028 | 219.1107 | [M-H]^-^ | 2.205 | 184.0355, 146.0083 |
| 6. | Kinsendioside A | C_16_H_26_O_13_ | 6.09 | 471.1344 | 426.1373 | [M+HCOO]^-^ | -0.162 | 425.1464, 341.1089, 179.0522 |
| 7. | Glucosyringic acid | C_15_H_20_O_10_ | 7.59 | 359.0981 | 360.1056 | [M-H]^-^ | 2.247 | 197.0391, 182.0562, 166.0612 |
| 8. | Gastrodin | C_13_H_18_O_7_ | 10.15 | 331.1031 | 286.1053 | [M+HCOO]^-^ | 2.209 | 161.0445, 145.0243, 123.0187 |
| 9. | Glucosylrutin | C_33_H_40_O_21_ | 12.53 | 771.1991 | 772.2062 | [M-H]^-^ | 1.641 | 609.1714, 301.0389 |
| 10. | Kinsenoside | C_10_H_16_O_8_ | 13 | 263.0773 | 264.0845 | [M-H]^-^ | -0.657 | 179.0562, 161.0445 |
| 11. | Trans-3-coumaric acid | C_9_H_8_O_3_ | 13.54 | 163.0392 | 164.0473 | [M-H]^-^ | 1.591 | 103.0023, |
| 12. | Quercetin 3-rutinoside-7-glucoside | C_33_H_40_O_18_ | 14.18 | 771.1968 | 772.2062 | [M-H]^-^ | -1.367 | 463.1703, 301.0060 |
| 13. | Quercetin 3,7-diglucoside | C_27_H_30_O_17_ | 14.31 | 625.1411 | 626.1483 | [M-H]^-^ | 1.799 | 300,0586 271.0685 |
| 14. | Rutin | C_27_H_30_O_16_ | 14.48 | 609.1471 | 610.1534 | [M-H]^-^ | 3.413 | 301.0786, 300.0951 |
| 15. | *p*-Hydroxycinnamic acid | C_9_H_8_O_3_ | 15.51 | 163.0391 | 164.0473 | [M-H]^-^ | 0.885 | 115.0499 |
| 16. | Kaempferol 3,7-diglucoside | C_27_H_30_O_16_ | 16.93 | 609.1459 | 610.1534 | [M-H]^-^ | 1.525 | 301.0278, 271.0245 |
| 17. | Isoquercitrin | C_21_H_20_O_12_ | 17.52 | 463.0881 | 464.0473 | [M-H]^-^ | 2.219 | 301.0389, 271.0381, 255.0226 |
| 18. | Quercimeritrin | C_21_H_20_O_12_ | 17.53 | 487.0845 | 464.0473 | [M+Na]^+^ | -0.466 | 303.0499， 287.2210 |
| 19. | Kaempferol-rutoside | C_27_H_30_O_15_ | 20.25 | 593.1513 | 594.1585 | [M-H]^-^ | 2.046 | 284.0525, 229.0460 |
| 20. | Orientin | C_21_H_22_O_12_ | 21.00 | 447.0933 | 448.1006 | [M-H]^-^ | 1.436 | 357.0687, 327.0589, 297.0479 |
| 21. | Naringin | C_27_H_32_O_14_ | 21.05 | 581.1863 | 580.1792 | [M+H]^+^ | -0.348 | 273.1671， 153.0658 |
| 22. | Isorhamnetin, 3,4'-diglucoside | C_28_H_32_O_17_ | 21.39 | 639.1567 | 640.1640 | [M-H]^-^ | 1.728 | 519.1154, 477.1751 |
| 23. | Narcissoside | C_28_H_32_O_16_ | 21.41 | 623.1616 | 624.1690 | [M-H]^-^ | 1.539 | 300.0275, 271.0248 |
| 24. | Astragalin | C_21_H_22_O_12_ | 21.89 | 447.0933 | 448.1006 | [M-H]^-^ | 2.389 | 301.0285, 179.0340 |
| 25. | Isorhamnetin 7-glucoside | C_22_H_22_O_12_ | 22.07 | 477.1036 | 478.1111 | [M-H]^-^ | 1.860 | 315.0582, 300.0380, 271.0382 |
| 26. | Naringenin | C_15_H_12_O_5_ | 23.08 | 273.0756 | 272.0685 | [M-H]^-^ | -0.476 | 153.0657, 119.0252 |
| 27. | Biochanin A | C_16_H_12_O_5_ | 24.62 | 285.0755 | 284.0685 | [M+H]^+^ | -0.877 | 253.1430, 229.1432 |
| 28. | Quercetin-7-O-glucoside | C_21_H_20_O_12_ | 26.91 | 463.0856 | 464.0955 | [M-H]^-^ | -3.244 | 301.1053, 179.0340, 151.0390 |
| 29. | Methyl Feruloylquinate | C_18_H_22_O_9_ | 27.26 | 381.1190 | 382.1264 | [M-H]^-^ | 2.575 | 193.0499, 117.0332 |
| 30. | Kaempferol | C_15_H_10_O_6_ | 27.70 | 285.0394 | 286.0477 | [M-H]^-^ | 0.230 | 257.0048, 185.0558 |
| 31. | Isorhamnetin | C_16_H_12_O_7_ | 28.12 | 315.0507 | 316.0583 | [M-H]^-^ | 2.415 | 163.0389, 151.0502 |
| 32. | Emodin | C_15_H_10_O_5_ | 28.64 | 271.0601 | 270.0528 | [M+H]^+^ | -0.037 | NA |
| 33. | Batatasin III | C_15_H_16_O_3_ | 28.93 | 243.1028 | 244.1099 | [M-H]^-^ | 4.850 | 181.0721, 106.0397 |
| 34. | Formononetine | C_16_H_12_O_4_ | 29.73 | 269.0810 | 268.0736 | [M+H]^+^ | 0.575 | 213.1481, 197.0810 |
| 35. | Wogonin | C_16_H_12_O_5_ | 30 | 2850759 | 284.0685 | [M+H]^+^ | 0.421 | 270.0677， 252.0291 |

**Table S3** Contents of representative ACR-reactive compounds in A. roxburghii extract Compound.

| **NO.** | **compound** | **Calibration equation** | **R^2^** | **Content** | **RSD (%)** |
| --- | --- | --- | --- | --- | --- |
| 1. | Rutin | y = 49109x - 4603 | 0.9982 | 30.78 ± 0.28 μg/g extract | 0.91 |
| 2. | NAR | y = 13730x + 5666.1 | 0.9961 | 209.44 ± 3.81 μg/g extract | 1.82 |
| 3. | ISO | y = 14141x - 170.34 | 0.9993 | 2.64 ± 0.05 μg/g extract | 1.71 |

Note: Values are expressed as mean ± SD (n = 3).

**Table S4** Typical compounds identified of the ISO-ACR adducts by UPLC-Q-Orbitrap HRMS/MS in ISO Group.

| **NO.** | **compound** | **Formula** | **RT (min)** | **Measured**  **(m/z)** | **Expected**  **m/z** | **Adductsion** | **Error**  **(ppm)** |
| --- | --- | --- | --- | --- | --- | --- | --- |
| 1. | ISO | C_16_H_12_O_7_ | 9.98 | 315.0510 | 316.0583 | [M-H]^-^ | 3.27 |
| 2. | ISO-momo-ACR | C_19_H_18_O_8_ | 10.22 | 371.0772 | 372.0845 | [M-H]^-^ | 2.95 |
| 3. | ISO-di-ACR | C_22_H_20_O_9_ | 9.76 | 427.1036 | 428.1170 | [M-H]^-^ | 2.86 |
| 4. | ISO-tri-ACR | C_25_H_24_O_10_ | 6.48 | 483.1294 | 484.1369 | [M-H]^-^ | 2.52 |
